# Editorial: Hemostasis in ECMO and VAD

**DOI:** 10.3389/fmed.2019.00143

**Published:** 2019-06-25

**Authors:** Jun Teruya, M. Patricia Massicotte, Barbara Zieger

**Affiliations:** ^1^Baylor College of Medicine, Texas Children's Hospital, Houston, TX, United States; ^2^Department of Pediatrics, University of Alberta, Stollery Children's Hospital, Edmonton, AB, Canada; ^3^Division of Pediatric Hematology and Oncology, Department of Pediatrics and Adolescent Medicine, Faculty of Medicine, Medical Center-University of Freiburg, University of Freiburg, Freiburg, Germany

**Keywords:** extracorporeal membrane oxygenation (ECMO), ventricular assist device (VAD), bleeding, neonate, novel surface, acquired von Willebrand syndrome

The indication for extracorporeal membrane oxygenation (ECMO) has been expanded in the recent years. The new indication includes ECMO cardiopulmonary resuscitation, known as ECPR, and sepsis. Since patients on ECMO are usually critically ill, coagulation derangement such as disseminated intravascular coagulation (DIC) is not uncommon. Hemostasis in ECMO is extremely complex; however it provides us a great model to understand the mechanism of overall hemostasis and anticoagulation in the presence of an artificial surface. How can we improve survival and decrease the incidence of bleeding and thrombosis? In this research topic, Hemostasis in ECMO and ventricular assist device (VAD), we included topics of activation mechanism of hemostasis, acquired von Willebrand syndrome (AVWS), neonatal ECMO, novel surface of tubes to prevent clot formation, and hemolysis.

Doyle and Hunt described the overall activation mechanism of hemostasis in ECMO. It involves not only coagulation factors and platelets, but also neutrophil adhesion, release of tissue factor and cytokines from monocytes, fibrinolysis and complement activation. They discuss cell-based coagulation activation which involves the extrinsic pathway and contact activation pathway (= intrinsic pathway) separately.

Van Ommen et al. discussed neonatal ECMO from developmental hemostasis. The overall hemostasis among neonates is different from older infants. They clearly explain the challenges of anticoagulation due to the immature hemostatic system and prevention and management of hemorrhage and thrombosis in neonatal ECMO. Unfractionated heparin is most commonly used as anticoagulant; however, the “best” monitoring technique of its anticoagulant effect remains to be determined.

Hensch et al. described different scenarios of ECMO. Some ECMO cases are unique and informative from a coagulation standpoint. They presented 4 cases; persistently high antithrombin levels throughout the course and the requirement for less heparin dose and no circuit change for 72 days; pulmonary hemorrhage managed by nebulizer with tranexamic acid or recombinant activated factor VII; sepsis induced DIC and purpura fulminans successfully managed by therapeutic plasma exchange; and a patient with respiratory failure resistant to heparin anticoagulation managed by bivalirudin.

Rauch et al. discussed that AVWS occurs in patients with continuous flow (CF)-VADs and ECMO. Characteristically for AVWS is the loss of the high molecular weight (HMW)-multimers which leads to reduced von Willebrand factor (VWF) collagen binding activity (VWF:CB), ristocetin cofactor activity (VWF:RCo), and VWF:Activity (VWF:Ac), respectively. This causes a decrease of the VWF:CB/VWF-Antigen (VWF:CB/VWF:Ag)-ratio, VWF:RCo/VWF-Antigen (VWF:RCo/VWF:Ag)-ratio, and VWF:Ac/VWF-Antigen (VWF:Ac/VWF:Ag)-ratio, respectively. Clinical implications of AVWS in VAD- and ECMO patients were reported and possible therapy was described. In addition, they discussed the data collected from a swine CF-VAD model which demonstrated that changes in pulsatility regimen can be pivotal to modulate VWF multimerization in plasma in high shear condition. Acute changes of pulse pressure induced a release of new VWF HMW-multimers from Weibel Palade Bodies.

Valladolid et al. described VWF, free hemoglobin, and thrombosis in ECMO. It is known that intravascular hemolysis causes increased levels of plasma free hemoglobin. It is associated with renal damage, hemostatic derangement, and higher mortality rate. They untangled the mechanism of thrombotic tendency due to VWF mediated platelet adhesion and thrombus formation on a surface-adsorbed fibrin(ogen) under high shear stress.

Ontaneda and Annich reviewed the development of both present and future novel surface in ECMO circuits. Systemic anticoagulation alone causes high risk for bleeding if excessive doses are administered and thrombosis if not enough anticoagulation is used. Circuits may be coated by biomimetic surfaces such as heparin and nitric oxide, and biopassive surfaces such as phosphorylcholine, poly-2-methoxyethlacrylate, and fluid-repellent surfaces. Lastly *in vitro* and *in vivo* endothelialization is discussed. However, all coating materials have advantages and disadvantages. Authors conclude that in order to achieve ultimate goal, i.e., less bleeding and thrombotic complications during ECMO, clinicians, basic scientists, and industry need to work together.

## Author Contributions

JT and BZ wrote the text. MM reviewed and edited the text.

### Conflict of Interest Statement

The authors declare that the research was conducted in the absence of any commercial or financial relationships that could be construed as a potential conflict of interest.

